# Cell atlas of the immune microenvironment in gastrointestinal cancers: Dendritic cells and beyond

**DOI:** 10.3389/fimmu.2022.1007823

**Published:** 2022-11-24

**Authors:** Yinuo Wang, Ting Yang, Huan Liang, Mi Deng

**Affiliations:** ^1^ Peking University International Cancer Institute, Peking University Health Science Center, Peking University, Beijing, China; ^2^ School of Basic Medical Sciences, Peking University Health Science Center, Peking University, Beijing, China; ^3^ Peking University Cancer Hospital and Institute, Peking University Health Science Center, Peking University, Beijing, China

**Keywords:** dendritic cells, T cells, gastrointestinal cancer, scRNA-seq, transcriptome

## Abstract

Gastrointestinal (GI) cancers occur in the alimentary tract and accessory organs. They exert a global burden with high morbidity and mortality. Inside the tumor microenvironment, dendritic cells (DCs) are the most efficient antigen-presenting cells and are necessary for adaptive immune responses such as T and B-cell maturation. However, the subsets of DCs revealed before were mostly based on flow cytometry and bulk sequencing. With the development of single-cell RNA sequencing (scRNA-seq), the tumor and microenvironment heterogeneity of GI cancer has been illustrated. In this review, we summarize the classification and development trajectory of dendritic cells at the single-cell level in GI cancer. Additionally, we focused on the interaction of DCs with T cells and their effect on the response to immunotherapy. Specifically, we focused on the newly identified tumor-infiltrating dendritic cells and discuss their potential function in antitumor immunity.

## Introduction

Gastrointestinal carcinoma refers to malignancies that occur in the alimentary tract and accessory organs. It consists of six main types of cancer: oesophageal cancer (OC), gastric cancer (GC), liver cancer (LC), gallbladder and biliary tract cancer (BTC), pancreatic cancer (PC) and colorectal cancer (CRC) (1). There were 5.09 million new cases of GI cancer and 3.61 million related deaths, accounting for 26.4% of the worldwide cancer incidence and 36.3% of all cancer-related deaths, respectively (2). Additionally, these numbers are on the upward trend compared to the epidemiological data before (3).

At present, the main treatments for GI cancers include surgery, chemotherapy, radiotherapy and targeted therapy, and the rise of immunotherapy improves the ways against the tumors and has gradually become the first-line therapy in GI cancer. Apart from pancreatic cancer, most clinical trials of GI cancer combine the anti-PD-1 antibody with sequential chemotherapy or targeted therapy to create new therapeutic paradigms (**Supplementary Table 1a**). The overall response rate (ORR) ranged from 26.7%-76.7%. Unfortunately, in pancreatic cancer, immune checkpoint blockade (ICB) therapy fails (ORR is almost 0%) and does not prolong the survival time compared with chemotherapy (4–7). Although ICB combined with chemotherapy has a higher ORR than targeted therapy, approximately half of patients still cannot benefit from it due to nonresponse, drug resistance, recurrence or disease progression. The overall resistance to ICB therapy is 11%-71% across all tumor types (8). These data from clinical trials indicate that releasing only T-cell brakes cannot fully eliminate tumors. To elicit whole-body immune activation and long-lasting immune memory, novel therapy methods need to be developed.

Dendritic cells (DCs), as the most efficient antigen-presenting cells, bridge the innate and adaptive immune systems. In the tumor microenvironment, DCs represent a heterogeneous group. Single-cell RNA sequencing can identify new DC subpopulations. Additionally, new insights provided by the single-cell transcriptome and spatial transcriptome will likely reveal a plethora of new immunotherapeutic interventions targeting specific DC subsets or their products for the treatment of a variety of human disorders, including cancers. In this review, we focus on high-resolution data on dendritic cells in GI cancer.

### Canonical development and traditional classification of DCs

Hematopoiesis gives rise to most immune cells. The classical three subtypes of DCs, monocyte-derived dendritic cells (Mo-DC), conventional dendritic cells (cDC) and plasmacytoid dendritic cells (pDC), all come from common dendritic progenitors (CDPs), common monocyte progenitors (cMoPs) (9) and IL-7R^+^ lymphoid progenitor cells (10). In one way, cMoPs give rise to CD14^+^ or CD16^+^ monocytes. When circulating monocytes encounter antigens, they differentiate into Mo-DCs and migrate to tissues later (11). In another way, the CDPs come to pre-pDCs and pre-cDCs. cDCs have two subtypes, type 1 (cDC1) and type 2 (cDC2), marked by CLEC9A^+^/CD141^+^/XCR1^+^ and CLEC10A^+^/SIRPα^+^/CD1c^+^ expression, respectively. cDCs have superior antigen presentation capacity. cDC1s present antigens to CD8^+^ T cells by MHC-I/TCR interactions, and cDC2s present antigens to CD4^+^ T cells by MHC-II/TCR interactions. Additionally, cDC1s cross-presented tumor-associated antigen (TAA) or tumor-specific antigen (TSA) to generate antigen-specific cytotoxic T cells is crucial in antitumor immunity (12). Furthermore, pDCs have a rounded shape that resembles plasma cells. Marked by CD123 in human, pDCs function during viral infection. They produce type I interferon upon stimulation with toll-like receptor (TLR) 7/9 (13).

DCs are the most potent antigen-presenting cells (APCs). They have four main functions, phagocytosis, antigen presentation, costimulatory/inhibitory ability and cytokine secretion ability, to regulate immunity. As professional phagocytes, DCs have separate pathways to process endogenous and exogenous antigens (14–16). After antigen uptake, DCs matured. They upregulate costimulatory molecules, such as cluster of differentiation 80/86 (CD80/86) and inducible T-cell costimulatory ligand (ICOSL), to provide the second signal of T-cell activation and proliferation. In addition, they secrete proinflammatory cytokines, such as interleukin-12 (IL-12), to promote differentiation from Th0 to Th1 (17) and tumor necrosis factor-alpha (TNF-α) to induce tumor cell apoptosis (18). Recently, a subset of DCs, CD103^+^DCs, have been considered to be crucial for trafficking to the lymph nodes and activating CD8^+^ T cells (19). Additionally, anti-PD-L1 blockade requires CD103^+^ DCs to promote but only have a partial response. Only when combined with FLT3L and poly I:C therapy can anti-PD-L1 blockade efficiently reduce tumors (20). Additionally, another study using an anti-PD-1 antibody and a multipeptide vaccine found that PD-1^+^ DCs decreased and memory precursor CD8^+^ T cells were upregulated (21). Altogether, these studies indicate the key subclusters of DCs in the response to immunotherapy and their regulation of memory CD8^+^ T cells.

### The updated taxonomy, ontogeny and new functions of DCs defined by scRNA-seq

With high-resolution sequencing technology at the single-cell level, novel clusters of DCs were discovered, and taxonomy was updated. First, cDC1 s and cDC2s come from the common dendritic cell progenitor (cDC progenitor), characterized by CD34^int^CD100^+^ (22). Moreover, a novel DC subcluster, AXL^+^ DCs characterized by AXL and SIGLEC6, was first identified in the peripheral blood of humans (23). AXL^+^DCs have a spectrum gene signature consisting of pDCs and cDCs, which indicates that they have the ability to give rise to both of them. Additionally, AXL^+^DCs were also found in the cord blood, which reveals their origin (24). However, they are more similar to cDCs in the adult cord clood than pDCs in the peripheral blood transcriptionally. Furthermore, by inhibiting AXL receptor tyrosine kinase, Li et al. found that cDCs increase type I interferon secretion and enhance the proliferation of TCF1^+^PD1^+^CD8^+^ T cells. This pathway sensitive the anti-PD1 blockade and restored the response (25).

Moreover, Rudensky et al. identified two functionally distinct cDC2 subclusters, T-bet^+^cDC2s and T-bet-cDC2s, in mice (26). T-bet^+^cDC2s have an anti-inflammatory profile, while T-bet^-^cDC2s show pro-inflammatory characteristics, secreting more TNF-α and IL-6 than T-bet^+^cDC2s. The researchers also validated that siglec-H^+^ cells have the progenitor nature to give rise to these cDC2 subclusters within the spleen. And in human, CD1c^lo^CLEC10A^–^CLEC4A^hi^ cDC2s and CD1c^+^CLEC10A^+^CLEC4A^lo^ cDC2s are counterparts of T-bet^+^cDC2s and T-bet^-^cDC2s in mice. Moreover, T-bet^+^cDC2s and CD1c^lo^CLEC10A^-^CLEC4A^hi^ cDC2s in the peripheral blood of mice and human respectively are absent consistently.

Recently, DCs were also found to maintain the exhaustion state in lymphoid or nonlymphoid tissue, which is vital during T-cell function (27, 28). Dähling et al. found that cDC1s prevent the overactivation of the precursors of exhausted T(Tpex) cells by providing a CCL21-dependent niche. They control their differentiation to exhausted T cells to balance the exhaustion state in the body (29). In addition, Schenkel et al. discovered that cDC1s helped tumor-specific CD8^+^ T cells, TCF-1^+^CD8^+^ T cells, to proliferate and differentiate into a heterogeneous population and thus reduced tumor burden (30). These studies revealed new insights into the contribution of DCs to immunity.

### Phenotypic alterations and novel tumor-infiltrating DCs identified by scRNA-seq

Although numerous potential stimulatory signals for DCs exist in the TME, many tumors also contain abundant amounts of immunosuppressive cytokines, such as IL-10 (31, 32). Orsini et al. found that colorectal cancer patients exhibited an impaired capacity to generate immature DCs from blood monocytes and lower expression levels of the costimulatory marker CD40 (33). Studies have found that the immunosuppressive chemokine CCL2 produced by tumor cells induces the autocrine secretion of lipocalin 2 (LCN2) and cooperatively generates immunoregulatory DCs (regDCs) with decreased HLA-DR expression and increased PD-L1 expression (34). In addition, the circulating pDCs recruited into the tumor microenvironment are characterized by decreased expression of costimulatory molecules and a reduced ability to produce type I interferons. Additionally, Li et al. found that pDCs played a potential role in recruiting Tregs, and both of them participate in the immunosuppressive microenvironment of GI cancer (35). Liu et al. also demonstrated that ICOS^+^ Tregs and pDCs predict a poor prognosis of gastric cancer (36). However, Abolhalaj et al. found that the myeloid/plasmacytoid dendritic cell ratio (mDC/pDC) was elevated in tonsillar cancer (37). Therefore, the landscape of tumor-infiltrating DCs and their functions need to be clarified.

Single-cell RNA sequencing has promoted the precise understanding of the tumor microenvironment. In **Supplementary Table 1b**, we summarize some high-quality single-cell RNA-seq data of GI cancers based on human tumor sample sequencing. In general, the ratio of DCs and T cells ranges from 1:5~1:12 (38–41). The proportion of mDCs is higher than that of pDCs, but the study did not give an accurate number (38). Three groups of DCs, cDC1s (highly expressed CLEC9A/BATF3), cDC2s (highly expressed CD1C/CLEC10A), and plasmacytoid DCs (highly expressed LILRA4), were detected in tumor and adjacent tissues.

Furthermore, the subtypes of tumor-infiltrating DCs are conserved across GI cancers. Recently, a novel tumor-infiltrating DC that highly expresses CCL19/LAMP3/CCR7 was identified. LAMP3^+^ cDCs were first identified by Zhang et al. (42) in hepatocarcinoma and is a kind of tumor-infiltrating DC that arises from cDC1 and cDC2. LAMP3^+^ cDCs were found in 15 different cancer types, which demonstrates that they have a broad appearance in tumors (43). They have the ability to migrate to hepatic lymph nodes because of high CCR7 expression. Apart from nasopharyngeal cancer and pancreatic adenocarcinoma, LAMP3^+^ cDCs preferentially come from cDC1s, which highly express IL12B and BTLA. Moreover, cDC2-derived LAMP3^+^ cDCs showed high expression of CCL17. Although these two kinds of LAMP3^+^ cDCs have distinct gene signatures, both of them have the capacity to induce Treg differentiation and recruitment. Additionally, the upregulated expression of PD-L1 and PD-L2 in LAMP3^+^ cDCs was consistent. And LAMP3^+^DCs were predicted to interact with PD-1 on Tregs, central memory T cells (CD4^+^ T cells highly expressing IL7R and TCF7), and effector memory T cells (CD8^+^ T cells highly expressing SELL GZMK) to regulate multiple kinds of T cells (42). Additionally, CCR7- and LAMP3-upregulated DCs were also detected in colorectal tumors (44). Altogether, these facts indicated that LAMP3^+^ cDCs are newly identified regulatory-like dendritic cells in the TME.

Moreover, different from the previous theory mentioned above, scRNA-seq further predicts the ligand−receptor interaction between DCs and T cells (42). cDC1s (DC-CLEC9A) have the ability to present antigens to CD4^+^T cells and cDC2 (DC-CD1c) are able to interact with CD8^+^T cells. Moreover, Cheng et al. performed a pan-cancer scRNA-seq and found that the proportion of cDC2s was higher than that of cDC1s in tumors. Some research found that ascites from hepatocarcinoma patients were enriched with DCs expressing FCER1A (DC-FCER1A) (42).

### Spatial distribution of dendritic cells in GI cancer

Apart from cell clustering at the single-cell level, the location of dendritic cells in the tumor is also crucial for their biological behavior. In oesophageal cancer, PD-L1^+^ or PD-L1^-^ DCs were nearest to PD-L1^+^ or PD-L1^-^ tumor cells, respectively (45). The closer distance between these two cells is correlated with better overall survival and progression-free survival. Moreover, in gastric cancer, DCs are sparse and scattered in the tumor (46). In hepatocarcinoma, cDCs were found to be significantly enriched in the normal regions instead of the tumor regions by spatial transcriptomics. Between the normal region and tumor region, there is a complete capsule that blocks the immune cells from entering the tumor (47). Furthermore, pDCs highly marked with BDCA-2 are located in the tertiary lymphoid structure (TLS) and correlate with prolonged survival in colorectal cancer (48). These data indicate that anti-tumor cDCs are not enriched in the tumor and that pro-tumor pDCs may contribute to tumor progression in GI cancer.

Additionally, a kind of dendritic cell termed follicular dendritic cells (FDCs) specifically originate from stromal cells located in the primary lymphoid organs, secondary lymphoid organs and TLSs (49). FDCs mainly induce a humoral response, unlike the cDCs and pDCs mentioned before. They mainly secrete C-X-C motif chemokine ligand 13 (CXCL13) to recruit B cells to B-cell follicles and assist them in differentiating into plasma cells and memory cells. Unlike MHC-TCR antigen presentation in T-cell activation, FDCs present unprocessed antigen to B cells with immune complexes (50). FDCs, B cells, and T cells dominantly form the tertiary lymphoid structure, and TLSs are correlated with better overall survival and progression-free survival (51–53).

However, the spatial information of DCs is relatively limited. Many studies utilizing spatial transcriptomics have not paid much attention to dendritic cell distribution and its potential role in presenting antigens in tumors (54, 55). Therefore, it is necessary to identify the distribution of DCs in GI cancers since it is vital for antigen presentation behavior.

### The difference between clinical samples and tumors from mouse models

**Figure 1:**
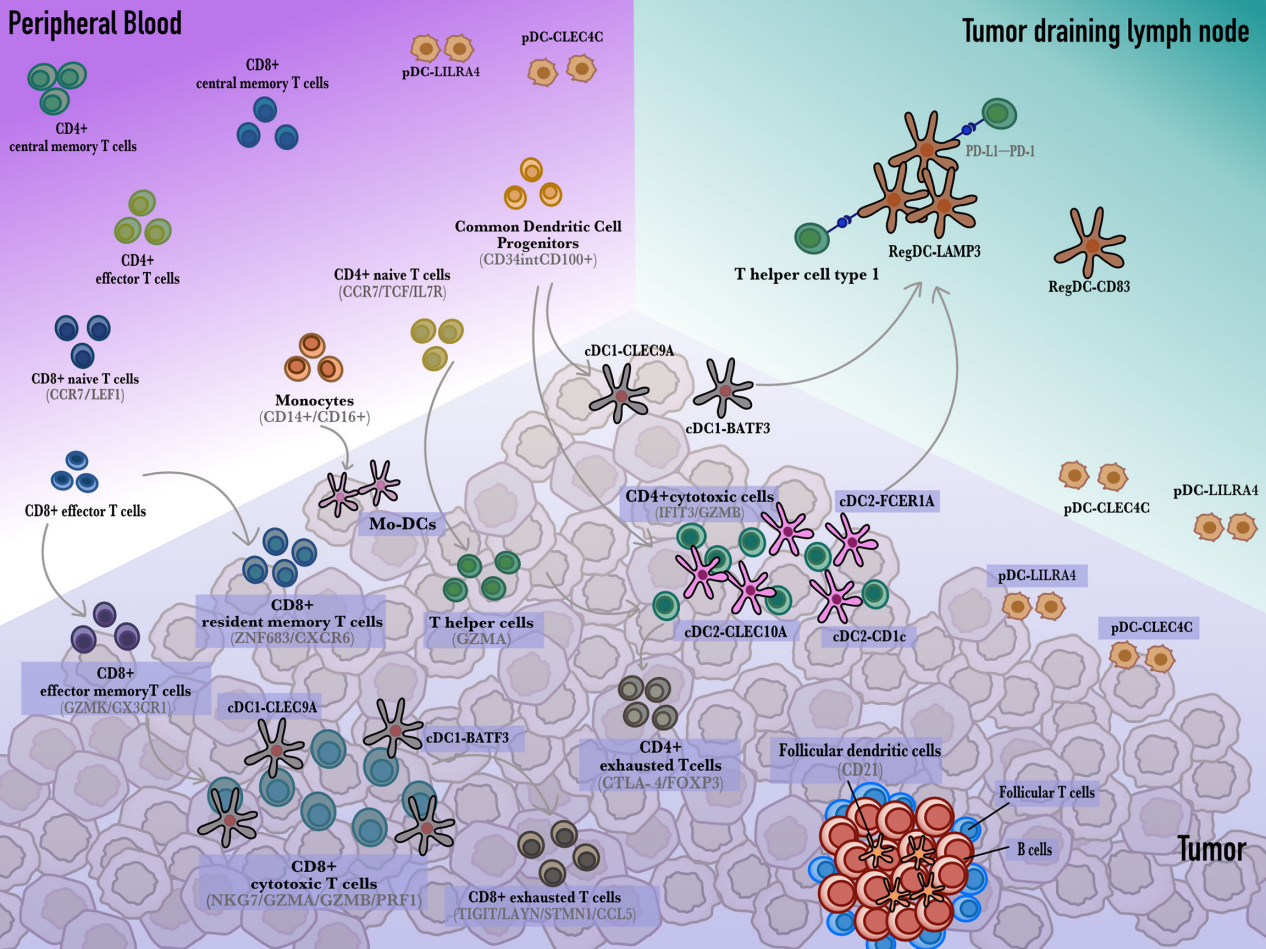
The developmental trajectory of dendritic cells and T cells. Monocytes circulate in the peripheral blood and differentiate into dendritic cells that sample and cargo tumor antigens. In the tumor draining lymph nodes, dendritic cells present antigens to CD4^+^ or CD8^+^ T cells to activate them into cytotoxic T cells. Additionally, cDCs also interact with resident CD8^+^ T cells to develop into CD8^+^ cytotoxic T cells. Besides, cDC1s and cDC2s give rise to regulatory LAMP3^+^ DC which induce CD8^+^ T cells exhaustion through the PD-L1-PD-1 axis.

The single-cell transcriptome and spatial transcriptome have revealed the DC atlas of human (**Figure 1**). However, the mouse model is the main preclinical model used to study dendritic cells and the immune system. To compare the difference in dendritic cells and T cells between human clinical samples and mouse tumors in GI cancer, we summarized the scRNA-seq data of mouse models in **Supplementary Table 1b**. Obviously, the subcluster differs between human and mice. Zhao et al. sequenced BALB/C, C57BL/6, SCID and SCID-HT29 liver cancer mouse models by scRNA-seq. They identified six DC subclusters, some of which were consistent with human data. Undeniably, the representative gene LILRA4, which is highly expressed in pDCs in humans, did not exist in mice. Additionally, the remarkable LAMP3 gene expression in human regDCs was dismissed in the mouse (56). A similar situation occurred in the CRC/GC mouse model, in which the classification of DC cells was too broad (57, 58). Additionally, DC gene expression in esophageal squamous cell carcinoma mouse models mostly does not match that in human tumors (59). Guilliams et al. (60) systematically compared murine and human liver cells at the single-cell level and found that the subclusters were mostly conserved. However, the gene expression of cDC1 s and cDC2s is quite distinct. Altogether, there is some discrepancy in the gene expression of the main immune clusters between mice and humans. The diversity of mouse cancer models is limited and cannot represent the heterogeneity of clinical samples.

To address the problem mentioned above, humanized model technology is appealing. The humanized mouse model (humice) refers to human CD34^+^ cells/peripheral blood mononuclear cells (PBMCs) engrafted in severe combined immunodeficiency mice. Zhao et al. established the humice platform of liver cancer. Flow cytometry monitors at least twenty-one human immune subsets, including pDC and mDC, in peripheral blood and various cytokine secretion in sera for 8 weeks. Then, the patients’ tumors were implanted subcutaneously in the humice. They found that the therapeutic effect of pembrolizumab was significantly better than that of ipilimumab. There were obvious toxic and side effects in humice when using ipilimumab, which is consistent with the clinical information (61). Therefore, the humice system provides a platform to detect the interaction between human immune cells and better imitates the cancer immunity of humans.

### Therapeutic strategy of DC-based cancer treatment

DC-based immunotherapy mainly refers to DC-based cancer vaccines. DC vaccines are mostly based on Mo-DCs from patients, which are accessible and have a mature culture protocol. Monocytes can be induced to differentiate into DCs stimulated by IL-4 and GM-CSF and to mature by LPS (62–64), which is a convenient source to generate DC vaccines. Then, using tumor lysates or predicted personalized tumor antigens, mo-DCs are activated and transferred to the patients. There are ~80 phase I/II clinical trials to treat gastrointestinal cancer (**Supplemental Table 2**). DC vaccines are currently recommended to use a ‘prime-boost’ strategy. Traditional treatment modalities first induce immunogenic cell death (ICD), and subsequent DC vaccination can boost a stronger immune response (65). DC vaccines have successfully treated highly immunogenic cancer. Provenge is the first FDA-approved DC vaccine used to treat prostate cancer since 2010 (66). These attempts have elucidated the safety and response of DC vaccines elicited in patients. Generally, the strategy using DC vaccine is now recommended to combine chemotherapy or ICB therapy because the single ORR of DC vaccine did not exceed 15% (67).

Although some regimens do not target DCs directly, scRNA-seq detected changes in DCs contributing to a better response. In an MC38 CRC mouse model treated with anti-CD40 agonist therapy, CCL22^+^ cDC1s along with CCL5^+^ CD8 effector memory T cells were enriched, and an elevated cDC1 gene signature was correlated with longer overall survival (68). In another study, when undergoing anti-PD-1 or anti-CTLA-4 blockade therapy, CD8 effector memory T cells highly expressing GZMK and HSPA1A were also upregulated in patients, which the latter has not been annotated previously in liver cancer (69). However, scRNA-seq of patients or mouse models before and after treatment is limited, and more attention should be given to not only T cells.

## Conclusion

Currently, scRNA-seq has identified novel subclusters and the precise function of DCs. In summary, conventional dendritic cells (cDC1 and cDC2) are the most efficient antigen presenting cells in the tumor draining lymph nodes and tumor microenvironment. The immunotherapy response is correlated with the interaction of cDCs and tumor-specific T cells. However, cDC1s and cDC2s can also differentiate into a subset of regulatory DCs (LAMP3^+^ DCs) to hamper anti-tumor immunity. Therefore, the future strategy to develop novel DC vaccines is to elicit a CD8^+^ T-cell response and prevent it from being changed by the immunosuppressive tumor microenvironment, thus eliminating the tumor burden.

## Author contributions

YW collected literature data. YW, TY and HL conceived and wrote the manuscript. MD revised the manuscript. All authors contributed to the article and approved the submitted version.

## Funding

This work was supported by the Imported Scholar Project and Startup from Peking University Health Science Center (BMU2021YJ063 to MD), the Biotechnology Innovation Plan from Beijing Sungen Biomedical Technology Co., Ltd (2022066 to MD), and the Excellent Young Scientists Fund Program (overseas) from National Natural Science Fund (2021HY-7 to MD). The funder Beijing Sungen Biomedical Technology Co., Ltd was not involved in the study design, collection, analysis, interpretation of data, the writing of this article or the decision to submit it for publication.

## Conflict of interest

The authors declare that the research was conducted in the absence of any commercial or financial relationships that could be construed as a potential conflict of interest.

## Publisher’s note

All claims expressed in this article are solely those of the authors and do not necessarily represent those of their affiliated organizations, or those of the publisher, the editors and the reviewers. Any product that may be evaluated in this article, or claim that may be made by its manufacturer, is not guaranteed or endorsed by the publisher.
