# Supplementary material for: Cell atlas of the immune microenvironment in gastrointestinal cancers: Dendritic cells and beyond
Source: Front Immunol. 2022 Nov 24;13:1007823. doi: 10.3389/fimmu.2022.1007823 (PMC9729272; doi:10.3389/fimmu.2022.1007823)
Supplement: Supplementary file 1 [file Table_1.docx]

## Supplementary Table 1. Representative clinical trials and DC and T subclusters defined in scRNA-seq in GI cancer

## a. Collection of clinical trials rewriting the therapeutic paradigm in GI cancer

| Cancer type | Clinical trial | Interventions | Molecular targets | PFS/months  (A vs. B vs. C) | mOS/months  (A vs. B vs. C) | Overall response rate  (A vs. B vs. C) | Phase | NCT number | FDA status | Reference |
| --- | --- | --- | --- | --- | --- | --- | --- | --- | --- | --- |
| Oesophegeal cancer | First-line oesophageal Carcinoma Study With Chemo vs. Chemo Plus Pembrolizumab (MK-3475-590/KEYNOTE-590)-China Extension Study | A: Pembrolizumab plus chemotherapy  B: Placebo plus chemotherapy | PD-1 | 6.3 *vs* 5.8 | 12.4 *vs* 9.8 | 45% vs. 29.3% | Phase 3 | NCT03189719 | Approved | (1) |
|  | Nivolumab Combination Therapy in Advanced Esophageal Squamous-Cell Carcinoma (CheckMate-648) | A: Nivolumab plus chemotherapy  B: Nivolumab plus the monoclonal antibody ipilimumab  C: Chemotherapy | PD-1, CTLA-4 | 8.4 vs. 11.8 vs. 5.7 | 13.2 vs. 12.7 vs. 10.7 | 47% vs. 28% vs. 27% | Phase 3 | NCT03143153 | Approved | (2) |
|  | Sintilimab or Placebo With Chemotherapy in Esophageal Squamous Cell Carcinoma (ORIENT-15) | A: Sintilimab plus chemotherapy  B: Placebo plus chemotherapy | PD-1 | 7.2 vs. 5.7 | 16.7 vs. 12.5 | 75.5% vs. 56.9% | Phase 3 | NCT03748134 | Approved | (3) |
|  | A randomized, double-blind, phase III study of toripalimab versus placebo in combination with first-line chemotherapy for treatment naïve advanced or metastatic esophageal squamous cell carcinoma (ESCC) (JUPITER-06) | A: Toripalimab plus paclitaxel and cisplatin  B: Placebo plus paclitaxel and cisplatin | PD-1 | 5.7 vs. 5.5 | 17 vs. 11 | - | Phase 3 | NCT03829969 | Approved | (4) |
|  | Effect of Camrelizumab vs Placebo Added to Chemotherapy on Survival and Progression-Free Survival in Patients With Advanced or Metastatic Esophageal Squamous Cell Carcinoma: The ESCORT-1st Randomized Clinical Trial (ESCORT-1^ST^) | A: Camrelizumab plus paclitaxel and cisplatin  B: Placebo plus paclitaxel and cisplatin | PD-1 | 6.9 vs. 5.6 | 15.3 vs. 12 | 72.1% VS. 62.1% | Phase 3 | NCT03691090 | Approved | (5) |
| Gastric cancer | Sintilimab plus chemotherapy (chemo) versus chemo as first-line treatment for advanced gastric or gastroesophageal junction (G/GEJ) adenocarcinoma (ORIENT-16): First results of a randomized, double-blind, phase III study | A: Sintilimab plus chemotherapy  B: Placebo plus chemotherapy | PD-1 | 7.1 vs. 5.7 | 15.2 vs. 12.3 | 65.1% vs 58.7% | Phase 3 | NCT03745170 | Approved | (6) |
|  | First-line nivolumab plus chemotherapy versus chemotherapy alone for advanced gastric, gastro-esophageal junction, and esophageal adenocarcinoma (CheckMate 649): a randomized, open-label, phase 3 trial | A: Nivolumab plus chemotherapy  B: Chemotherapy | PD-1 | 7.7 vs. 6.9 | 14.4 vs. 11.1 | 60% vs 45% | Phase 3 | NCT02872116 | Approved | (7) |
|  | First-line pembrolizumab/placebo plus trastuzumab and chemotherapy in HER2-positive advanced gastric cancer: KEYNOTE-811 | A: Pembrolizumab plus trastuzumab and chemotherapy  A: Placebo plus trastuzumab and chemotherapy | PD-1, HER2 | Data to be reported at the time of the final analysis | | 74.4% VS. 51.9% | Phase 3 | NCT03615326 | Approved | (8) |
| Liver cancer | IMbrave150: Updated overall survival (OS) data from a global, randomized, open-label phase III study of atezolizumab (atezo) + bevacizumab (bev) versus sorafenib (sor) in patients (pts) with unresectable hepatocellular carcinoma (HCC) | A: Atezolizumab + bevacizumab  B: Sorafenib | PD-L1, VEGFR1-3, PDGFR-β, Flt-3, c-KIT | 6.8 vs. 5.3 | 19.2 vs. 13.4 | 35.4% vs. 13.9% | Phase 3 | NCT03434379 | Approved | (9) |
|  | Phase Ib Study of Lenvatinib Plus Pembrolizumab in Patients With Unresectable Hepatocellular Carcinoma | A: Lenvatinib plus Pembrolizumab | PD-1, VEGFR1-3, FGFR1-4, PDGFRα, KIT, RET | 9.3 | 22 | 46% | Phase 1b | NCT03006926 | Approved | (10) |
|  | A phase Ib study of lenvatinib (LEN) plus nivolumab (NIV) in patients (pts) with unresectable hepatocellular carcinoma (uHCC): Study 117. | A: Lenvatinib plus nivolumab | PD-1 | - | - | 76.7% | Phase 1b | NCT03418922 | Approved | (11) |
|  | Camrelizumab in Combination with Apatinib in Patients with Advanced Hepatocellular Carcinoma (RESCUE): A Nonrandomized, Open-label, Phase II Trial | A: Camrelizumab plus apatinib | PD-1，VEGFR2 | 20.3(estimated)  21.8(estimated) | 6.4(1 L)  5.5(2 L) | 46%(1 L)  35%(2 L) | Phase 2 | NCT03463876 | Approved | (12) |
| Biliary cancer | A phase 3 randomized, double-blind, placebo-controlled study of durvalumab in combination with gemcitabine plus cisplatin (GemCis) in patients (pts) with advanced biliary tract cancer (BTC): TOPAZ-1. | A: Durvalumab + GemCis  B: Placebo + GemCis | PD-1 | 7.2 vs. 5.7 | 12.8 vs. 11.5 | 26.7% vs. 18.7% | Phase 3 | NCT03875235 | Approved | (13) |
| Colorectal cancer | Health-related quality of life in patients with microsatellite instability-high or mismatch repair deficient metastatic colorectal cancer treated with first-line pembrolizumab versus chemotherapy (KEYNOTE-177): an open-label, randomized, phase 3 trial | A: Pembrolizumab  B: Chemotherapy | PD-1 | 16.5 vs. 8.2 | Data to be reported at the time of the final analysis | 43.8% vs. 33.1% | Phase 3 | NCT02563002 | Approved | (14) |
|  | Nivolumab in patients with metastatic DNA mismatch repair-deficient or microsatellite instability-high colorectal cancer (CheckMate 142): an open-label, multicenter, phase 2 study | A: Nivolumab | PD-1 | 14.3 | NR | 32% | Phase 2 | NCT02060188 | Approved | (15) |
|  | KEYNOTE-164: Pembrolizumab for patients with advanced microsatellite instability high (MSI-H) colorectal cancer. | A: Pembrolizumab | PD-1 | 4.1 | NR | 32% | Phase 2 | NCT02460198 | Approved | (16) |

## b. Single-cell RNA sequencing data for DC and T subclusters of GI cancer in human and mouse

|  | Human | | | | Mouse | | | | | The percentage of DC and T cells in the tumor |
| --- | --- | --- | --- | --- | --- | --- | --- | --- | --- | --- |
|  | Reference | Dendritic cells | CD4+ T cells | CD8+ T cells | Reference | Dendritic cells | | CD4+ T cells | CD8+ T cells |  |
| Pan-cancer | (17) | _ | c01(Tn); c02(CXCR5^+^pre-Tfh);  c03(ADSL^+^Tn); c04(IL7R^-^Tn);  c05(TNF^+^T); c06(AREG^+^Tm);  c07(TIMP1^+^Tm); c08(CREM^+^Tm);  c09(CCL5^+^Tm); c10(CAPG^+^Tm);  c11(CAPG^+^CREM^-^Tm); c12(GZMK^+^Tem);  c13(Temra); c14(CCR6^+^Th17);  c15(IL26^+^Th17); c16(IL21^+^Tfh);  c17(IFNG^+^Tfh/Th1); c18(TNFRSF9^+^Treg);  c19(SIPR1^+^Treg); c20(TNFRSF9^+^Treg);  c21(ISG^+^Treg); c22(ISG^+^Th);  c23(NME1^+^CCR4^-^T);c24(NME1^+^CCR4^+^T); | c01(Tn); c02(IL7R^+^Tm);  c03(uncharacterized); c04(ZNF683^+^CXCR6^-^Tm);  c05(GZMK^+^early Tem); c06(GZMK^+^Tem);  c07(Temra); c08(KIR^+^EOMES^+^NK-like);  c09(KIR^+^TXK^+^NK-like); c10(ZNF683^+^CXCR6^+^Trm);  c11(GZMK^+^Tex); c12(terminal Tex);  c13(OXPHOS^-^Tex); c14(TCF7^+^Tex);  c15(ISG^+^CD8^+^T); c16(Tc17);  c17(NME1^+^T); | _ | _ | | _ | _ | _ |
|  | (18) | pDC-LILRA4;  cDC1-CLEC9A;  cDC2-CD1C; | _ | _ |  |  |  |  |  | _ |
| Oesophageal Cancer | (19) | DC-CLEC9A; DC-CLEC10A;  DC-LAMP3; DC-FCER1A;  DC-CLEC4C; | CD4-CCR7; CD4-TCF7;  CD4-CD40LG; CD4-IFIT3;  CD4-STMN1; CD4-FOXP3; | CD8-NKG7; CD8-HSPA1A;  CD8-GZMK; CD8-ZNF683;  CD8-CCL5; CD8-STMN1;  CD8-TIGIT; | (20) | pDC-SIGLECH;  tolerogenic dendritic cell (tDC)-BATF3;  cDC1-ITGAX;  cDC1-CLEC9A;  cDC2-CCR2;  cDC2-BST2; | | TN1, TN2, TN3 (SELL, TCF7, CCR7);  Th1-GZMB; Th2-GATA3;  Th17-IL17a; Treg-FOXP3; | TN1, TN2, TN3, TN4(IL7R, TCF7, CCR7);  TCM, TEM, TRM(BCL2, SELL, CD44); | DC:~4.3%  T: 51.42% |
| Gastric cancer | (21) | DC-PLD4 | CD4-CCR7; CD4-STAT4;  Treg-IL2RA; Treg-STAT3; | CD8-CD8A; CD8-GZMH;  CD8-GZMK; CD8- NKG7; | (22) | | DC-CD103;  DC-CD11b; | CD4-HAVCR2;  CD4-IL10/CTLA-4;  CD4-IL17a; | CD8-CTLA-4;  CD8-CD160/GZMB;  CD8-IFNG; | DC / T= ~1:12 |
|  | (23) | DC-pDCs; DC-mDCs | - | - |  |  |  |  |  | _ |
|  | (24) | DC-IL3RA/CLEC4C;  DC- CD83/CCR7/IL7R/ID2 | CD4-CCR7; CD4- SELL;  CD4-CD74; CD4-GZMA/CXCL13; | CTLs-naïve tumor(CCR7);  CTLs-effector normal CTLs(CD69);  CTLs-effector PBMC CTLs(CD8A/B);  CTLs-tumor effector(GZMA/B);  CTLs-tumor effector(NKG7); |  |  |  |  |  | _ |
|  | (25) | DC-activated; DC-migratory | Th-LMNA; Th-RPS26;  Th-RPLP2; Th-NKG7;  Th-TIGIT; Th-CXCR6;  Th-GNLY; Th-ICA1;  Treg-IL32; Treg-RPLP2;  Treg-CCL5; Treg-CXCL13; | CTL-NKG7; CTL-IL7R;  CTL-IFNG; CTL-GZMB;  CTL-PDCD1; |  |  |  |  |  | _ |
| Pancreatic cancer | (26) | pDC1; pDC2;  cDC1; cDC2_A; cDC2_B;  Langerhan_like_DC1; Langerhan_like_DC2;  Acitivated_DC1; Acitivated_DC2; | CD4-CCR7; CD4-FOXP3;  CD4-TNFRSF18; CD4-PDCD1;  CD4-ITGB1; CD4-ANXA2;  (other CD4+T relatively similar) | CD8-PRF1; CD8-TIGIT;  CD8-CCR6; CD8-IFITM3;  CD8-HAVCR2; CD8-CXCR4; | (27) | | pDC;  cDC-CD103 | _ | _ | _ |
| Hepatobiliary Cancer | (28) | _ | CD4-CCR7; CD4-FOXP3;  CD4-CTLA4; CD4-GZMA;  CD4-CXCL13; CD4-GNLY; | CD8-LEF1; CD8-CX3CR1;  CD8-SLC4A10; CD8-LAYN;  CD8-GZMK; | (29) | | cDC-RPL212;  cDC-CLEC10A;  cDC-CLECL9A;  cDC-RPL218;  cDC-CCR7;  pDC-SIGLECH; | _ | _ | _ |
|  | (30) | DC-CD1C; DC-FCER1A;  DC-CLEC9A; DC-LAMP3 | CD4-ANXA1; CD4-IL7R;  CD4-TCF7; CD4-CXCL13;  CD4-FOXP3; CD4-CCR7; | CD8-MKI67; CD8-IL7R;  CD8-CX3CR1; CD8-SELL;  CD8-GZMK; CD8-KLRD1;  CD8-PDCD1; CD8-SLC4A10; |  |  |  |  |  |  |
|  | (31) | _ | CD4-ANXA1; CD4-CD69;  CD4-CTLA-4; CD4-GZMA; | CD8-GZMA; CD8-GZMK; |  |  |  |  |  |  |
|  | (32) | DC-CLEC9A; DC-CD1C; | CD4–IL7R; Tregs–FOXP3 | CD8–GZMK; CD8–GZMB;  CD8–IL7R; CD8–MKI67; | (33) | | _ | Naïve CD4^+^T;  Effector CD4^+^T | Naïve CD8^+^T;  Effector CD8^+^T | DC / T = ~1: 14 |
|  | (34) | DC1-CLEC9A;  DC2-CD1C/CLEC10A;  DC3-CCL19/LAMP3/CCR7; | CD4−CCR7; CD4−IL2;  Treg-FOXP3; Treg-LAYN;  Cycling T; | CD8−CCR6; CD8−GZMK;  CD8−GZMH; CD8−XCL1;  CD8−NR4A1; CD8−CTLA4;  CD8−LAG3; |  |  |  |  |  | _ |
|  | (35) | _ | CD4−CCR7; CD4−CD69;  CD4−HS PA1A; CD4−IL7R;  CD4−CXCL13; CD4−MKI67−CXCL13; CD4−GZMK−HS PA1A; CD4−GZMH;  CD4−GNLY; CD4−FOXP3 | CD8−CCR7; CD8−HS PA1A;  CD8−IL7R; CD8−GZMK;  CD8−PDCD1; CD8−CXCL13;  CD8−MKI67−CXCL13; CD8−CD137; CD8−GNLY; CD8−NCR3; |  |  |  |  |  | _ |
| Colorectal cancer | (36) | _ | CD4−CCR7; CD4−ANXA1;  CD4−GNLY; CD4−TCF7;  CD4−CXCR6; CD4−CXCR5;  CD4−GZMK; CD4−IL23R;  CD4−CXCL13; CD4−FOXP3;  CD4−IL10; CD4−CTLA4; | CD8−LEF1; CD8−GPR183;  CD8−CX3CR1; CD8−GZMK;  CD8−CD6; CD8−CD160;  CD8−LAYN; CD8−SLC4A10; | (37) | | cDC; pDC; mDC; | T cells naïve; T cells proliferative; T cells regulatory;  T helper cells; CD4 effector memory; CD4 recently activated;  CD4 naïve memory; CD4 transitional memory; CD8 pre-exhausted; CD8 cytotoxic; CD8 effector memory; CD8 terminally exhausted; | | _ |
|  | (38) | pDC-LILRA4; cDC1-BATF3;  cDC2-CD1C; | CD4−CCR7; CD4−ANXA1;  CD4−GNLY; CD4−TCF7;  CD4−CXCR6; CD4−CXCR5;  CD4−GZMK; CD4−IL23R;  CD4−CXCL13; CD4−CTLA4; | CD8−LEF1; CD8−GPR183;  CD8−CX3CR1; CD8−GZMK;  CD8−CD6; CD8−CD160;  CD8−LAYN; |  |  |  |  |  | _ |
|  | (39) | DC-CD11c/CD83/CD209/HLA-DRA | CD4; CD8; Th1; Th2; Treg; CD8+ cytotoxic T cell; Effector T cell; Memory T-cell | |  |  |  |  |  | DC:7.17%  T: 40.96% |
|  | (40) | _ | cytotoxic T−GZMB/NKG7; naïve T cells−CCR7/TCF7;  exhausted CD4+ T helper cells−CD4/CXCL13;  HSP+ T cells- HSPA1A/HSPB1/HSPE1;  γδT−TRDC/TRGC; cycling T−CKS1B | |  |  |  |  |  | _ |

(The single maker and subclusters in the table are consistent with the original references and are representative gene markers of scRNA-seq)

**References:**

1. Sun JM, Shen L, Shah MA, Enzinger P, Adenis A, Doi T, et al. Pembrolizumab plus chemotherapy versus chemotherapy alone for first-line treatment of advanced oesophageal cancer (KEYNOTE-590): a randomised, placebo-controlled, phase 3 study. Lancet. 2021;398(10302):759-71.

2. Doki Y, Ajani JA, Kato K, Xu J, Wyrwicz L, Motoyama S, et al. Nivolumab Combination Therapy in Advanced Esophageal Squamous-Cell Carcinoma. The New England journal of medicine. 2022;386(5):449-62.

3. Shen L, Lu Z, Wang J, Shu Y, Kong L, Yang L, et al. Sintilimab plus chemotherapy versus chemotherapy as first-line therapy in patients with advanced or metastatic esophageal squamous cell cancer: First results of the phase III ORIENT-15 study. Annals of Oncology 2021;32 (suppl_5): S1283-S1346. 10.1016/annonc/annonc741.

4. Xu R, Wang F, Cui C, Yao J, Zhang Y, Wang G, et al. JUPITER-06: A randomized, double-blind, phase III study of toripalimab versus placebo in combination with first-line chemotherapy for treatment naive advanced or metastatic esophageal squamous cell carcinoma (ESCC). Annals of Oncology 2021;32 (suppl_5): S1040-S1075. 10.1016/annonc/annonc708.

5. Luo H, Lu J, Bai Y, Mao T, Wang J, Fan Q, et al. Effect of Camrelizumab vs Placebo Added to Chemotherapy on Survival and Progression-Free Survival in Patients With Advanced or Metastatic Esophageal Squamous Cell Carcinoma: The ESCORT-1st Randomized Clinical Trial. Jama. 2021;326(10):916-25.

6. Xu J, Jiang H, Pan Y, Gu K, Cang S, Han L, et al. Sintilimab plus chemotherapy (chemo) versus chemo as first-line treatment for advanced gastric or gastroesophageal junction (G/GEJ) adenocarcinoma (ORIENT-16): First results of a randomized, double-blind, phase III study. Annals of Oncology 2021;32 (suppl_5): S1283-S1346. 10.1016/annonc/annonc741.

7. Janjigian YY, Shitara K, Moehler M, Garrido M, Salman P, Shen L, et al. First-line nivolumab plus chemotherapy versus chemotherapy alone for advanced gastric, gastro-oesophageal junction, and oesophageal adenocarcinoma (CheckMate 649): a randomised, open-label, phase 3 trial. Lancet. 2021;398(10294):27-40.

8. Chung HC, Bang YJ, C SF, Qin SK, Satoh T, Shitara K, et al. First-line pembrolizumab/placebo plus trastuzumab and chemotherapy in HER2-positive advanced gastric cancer: KEYNOTE-811. Future Oncol. 2021;17(5):491-501.

9. Finn RS, Qin S, Ikeda M, Galle PR, Ducreux M, Kim TY, et al. Atezolizumab plus Bevacizumab in Unresectable Hepatocellular Carcinoma. The New England journal of medicine. 2020;382(20):1894-905.

10. Finn RS, Ikeda M, Zhu AX, Sung MW, Baron AD, Kudo M, et al. Phase Ib Study of Lenvatinib Plus Pembrolizumab in Patients With Unresectable Hepatocellular Carcinoma. J Clin Oncol. 2020;38(26):2960-70.

11. Kudo M, Ikeda M, Motomura K, Okusaka T, Kato N, Dutcus CE, et al. A phase Ib study of lenvatinib (LEN) plus nivolumab (NIV) in patients (pts) with unresectable hepatocellular carcinoma (uHCC): Study 117. Journal of Clinical Oncology. 2020;38(4_suppl):513-.

12. Xu J, Shen J, Gu S, Zhang Y, Wu L, Wu J, et al. Camrelizumab in Combination with Apatinib in Patients with Advanced Hepatocellular Carcinoma (RESCUE): A Nonrandomized, Open-label, Phase II Trial. Clin Cancer Res. 2021;27(4):1003-11.

13. Oh D-Y, He AR, Qin S, Chen L-T, Okusaka T, Vogel A, et al. A phase 3 randomized, double-blind, placebo-controlled study of durvalumab in combination with gemcitabine plus cisplatin (GemCis) in patients (pts) with advanced biliary tract cancer (BTC): TOPAZ-1. Journal of Clinical Oncology. 2022;40(4_suppl):378-.

14. Andre T, Amonkar M, Norquist JM, Shiu KK, Kim TW, Jensen BV, et al. Health-related quality of life in patients with microsatellite instability-high or mismatch repair deficient metastatic colorectal cancer treated with first-line pembrolizumab versus chemotherapy (KEYNOTE-177): an open-label, randomised, phase 3 trial. The Lancet Oncology. 2021;22(5):665-77.

15. Overman MJ, McDermott R, Leach JL, Lonardi S, Lenz HJ, Morse MA, et al. Nivolumab in patients with metastatic DNA mismatch repair-deficient or microsatellite instability-high colorectal cancer (CheckMate 142): an open-label, multicentre, phase 2 study. The Lancet Oncology. 2017;18(9):1182-91.

16. Le DT, Kavan P, Kim TW, Burge ME, Cutsem EV, Hara H, et al. KEYNOTE-164: Pembrolizumab for patients with advanced microsatellite instability high (MSI-H) colorectal cancer. Journal of Clinical Oncology. 2018;36(15_suppl):3514-.

17. Zheng L, Qin S, Si W, Wang A, Xing B, Gao R, et al. Pan-cancer single-cell landscape of tumor-infiltrating T cells. Science. 2021;374(6574):abe6474.

18. Cheng S, Li Z, Gao R, Xing B, Gao Y, Yang Y, et al. A pan-cancer single-cell transcriptional atlas of tumor infiltrating myeloid cells. Cell. 2021;184(3):792-809.e23.

19. Zheng Y, Chen Z, Han Y, Han L, Zou X, Zhou B, et al. Immune suppressive landscape in the human esophageal squamous cell carcinoma microenvironment. Nat Commun. 2020;11(1):6268.

20. Yao J, Cui Q, Fan W, Ma Y, Chen Y, Liu T, et al. Single-cell transcriptomic analysis in a mouse model deciphers cell transition states in the multistep development of esophageal cancer. Nat Commun. 2020;11(1):3715.

21. Kumar V, Ramnarayanan K, Sundar R, Padmanbahan N, Srivastava S, Koiwa M, et al. Single-cell atlas of lineage states, tumor microenvironment and subtype-specific expression programs in gastric cancer. Cancer Discov. 2021.

22. Nagaoka K, Shirai M, Taniguchi K, Hosoi A, Sun C, Kobayashi Y, et al. Deep immunophenotyping at the single-cell level identifies a combination of anti-IL-17 and checkpoint blockade as an effective treatment in a preclinical model of data-guided personalized immunotherapy. J Immunother Cancer. 2020;8(2).

23. Fu K, Hui B, Wang Q, Lu C, Shi W, Zhang Z, et al. Single-cell RNA sequencing of immune cells in gastric cancer patients. Aging (Albany NY). 2020;12(3):2747-63.

24. Sathe A, Grimes SM, Lau BT, Chen J, Suarez C, Huang RJ, et al. Single-Cell Genomic Characterization Reveals the Cellular Reprogramming of the Gastric Tumor Microenvironment. Clin Cancer Res. 2020;26(11):2640-53.

25. Jeong HY, Ham IH, Lee SH, Ryu D, Son SY, Han SU, et al. Spatially Distinct Reprogramming of the Tumor Microenvironment Based On Tumor Invasion in Diffuse-Type Gastric Cancers. Clin Cancer Res. 2021;27(23):6529-42.

26. Steele NG, Carpenter ES, Kemp SB, Sirihorachai V, The S, Delrosario L, et al. Multimodal Mapping of the Tumor and Peripheral Blood Immune Landscape in Human Pancreatic Cancer. Nat Cancer. 2020;1(11):1097-112.

27. Geller AE, Shrestha R, Woeste MR, Guo H, Hu X, Ding C, et al. The induction of peripheral trained immunity in the pancreas incites anti-tumor activity to control pancreatic cancer progression. Nat Commun. 2022;13(1):759.

28. Zheng C, Zheng L, Yoo JK, Guo H, Zhang Y, Guo X, et al. Landscape of Infiltrating T Cells in Liver Cancer Revealed by Single-Cell Sequencing. Cell. 2017;169(7):1342-56.e16.

29. Zhao Q, Molina-Portela MDP, Parveen A, Adler A, Adler C, E H, et al. Heterogeneity and chimerism of endothelial cells revealed by single-cell transcriptome in orthotopic liver tumors. Angiogenesis. 2020;23(4):581-97.

30. Zhang Q, He Y, Luo N, Patel SJ, Han Y, Gao R, et al. Landscape and Dynamics of Single Immune Cells in Hepatocellular Carcinoma. Cell. 2019;179(4):829-45.e20.

31. Ma L, Hernandez MO, Zhao Y, Mehta M, Tran B, Kelly M, et al. Tumor Cell Biodiversity Drives Microenvironmental Reprogramming in Liver Cancer. Cancer cell. 2019;36(4):418-30.e6.

32. Zhang M, Yang H, Wan L, Wang Z, Wang H, Ge C, et al. Single-cell transcriptomic architecture and intercellular crosstalk of human intrahepatic cholangiocarcinoma. J Hepatol. 2020;73(5):1118-30.

33. Hao X, Chen Y, Bai L, Wei H, Sun R, Tian Z. HBsAg-specific CD8(+) T cells as an indispensable trigger to induce murine hepatocellular carcinoma. Cell Mol Immunol. 2021;18(1):128-37.

34. Sun Y, Wu L, Zhong Y, Zhou K, Hou Y, Wang Z, et al. Single-cell landscape of the ecosystem in early-relapse hepatocellular carcinoma. Cell. 2021;184(2):404-21.e16.

35. Ma L, Wang L, Khatib SA, Chang CW, Heinrich S, Dominguez DA, et al. Single-cell atlas of tumor cell evolution in response to therapy in hepatocellular carcinoma and intrahepatic cholangiocarcinoma. J Hepatol. 2021;75(6):1397-408.

36. Zhang L, Yu X, Zheng L, Zhang Y, Li Y, Fang Q, et al. Lineage tracking reveals dynamic relationships of T cells in colorectal cancer. Nature. 2018;564(7735):268-72.

37. Nieto P, Elosua-Bayes M, Trincado JL, Marchese D, Massoni-Badosa R, Salvany M, et al. A single-cell tumor immune atlas for precision oncology. Genome Res. 2021;31(10):1913-26.

38. Zhang L, Li Z, Skrzypczynska KM, Fang Q, Zhang W, O'Brien SA, et al. Single-Cell Analyses Inform Mechanisms of Myeloid-Targeted Therapies in Colon Cancer. Cell. 2020;181(2):442-59.e29.

39. Zhang Y, Song J, Zhao Z, Yang M, Chen M, Liu C, et al. Single-cell transcriptome analysis reveals tumor immune microenvironment heterogenicity and granulocytes enrichment in colorectal cancer liver metastases. Cancer Lett. 2020;470:84-94.

40. Wang W, Zhong Y, Zhuang Z, Xie J, Lu Y, Huang C, et al. Multiregion single-cell sequencing reveals the transcriptional landscape of the immune microenvironment of colorectal cancer. Clin Transl Med. 2021;11(1):e253.
