# Supplementary material for: Cell atlas of the immune microenvironment in gastrointestinal cancers: Dendritic cells and beyond
Source: Front Immunol. 2022 Nov 24;13:1007823. doi: 10.3389/fimmu.2022.1007823 (PMC9729272; doi:10.3389/fimmu.2022.1007823)
Supplement: Supplementary file 2 [file Table_2.docx]

## Supplementary Table 2. DCs based clinical trials of gastrointestinal cancer

| NO. | NCT Number | Title | Status | Conditions | Interventions | Phases |
| --- | --- | --- | --- | --- | --- | --- |
| 1 | NCT03410732 | Dendritic Cell-based Immunotherapy in Treatment Gastric Cancer | Unknown status | Gastric Cancer | Biological: activated DCs\|Procedure: radical surgery only | Phase 2 |
| 2 | NCT02496273 | Phase I Trial of CEA Specific AAV-DC-CTL Treatment in Stage IV Gastric Cancer | Active, not recruiting | Gastric Cancer | Biological: CTL | Phase 1 |
| 3 | NCT01637805 | Clinical Safety and Preliminary Efficacy of AAV-DC-CTL Treatment in Stage IV Gastric Cancer | Unknown status | Stage IV Gastric Cancer | Biological: Antigen-specific cytotoxic T lymphocytes induced by dendritic cells infected by recombinant adeno-associated virus with CEA gene | Phase 1 |
| 4 | NCT01783951 | Study of S-1 Plus DC-CIK for Patients With Advanced Gastric Cancer | Completed | Gastric Cancer | Biological: DC-CIK\|Drug: S-1\|Drug: Cisplatin | Phase 1\|Phase 2 |
| 5 | NCT02602249 | Clinical Safety and Preliminary Efficacy of MUC1-DC-CTL Treatment in Stage IV Gastric Cancer. | Unknown status | Gastric Cancer | Biological: MUC1-gene-DC-CTL\|Biological: MUC1-peptide-DC-CTL | Phase 1 |
| 6 | NCT02504229 | DC-CIK In Combination With Chemotherapy ( Gio / Oxaliplatin or Cisplatin ) Versus First-line Chemotherapy for Locally Advanced Unresectable or Metastatic Gastric Adenocarcinoma Randomized Controlled Phase II Clinical Study of Treatment | Unknown status | Gastric Cancer | Biological: autologous dendritic cells co-cultured with cytokine-induced killer cells\|Drug: Gimeracil and Oteracil Porassium Capsules\|Drug: Oxaliplatin | Phase 2 |
| 7 | NCT00005956 | Biological Therapy in Treating Patients With Advanced Cancer | Completed | Breast Cancer\|Gastric Cancer\|Ovarian Cancer | Biological: HER-2/neu intracellular domain protein\|Biological: therapeutic autologous dendritic cells | Not Applicable |
| 8 | NCT02632201 | Immunotherapy Using Pluripotent Killer-Human Epidermal Growth Factor Receptor-2 (PIK-HER2) Cells for the Treatment of Advanced Gastric Cancer With Liver Metastasis | Unknown status | Liver Metastasis\|Gastric Cancer | Biological: PIK-HER2\|Biological: DC-PMAT | Phase 1\|Phase 2 |
| 9 | NCT03185429 | TSA-DC Vaccine in Treating Patients With Gastrointestinal Solid Tumor | Unknown status | Tumor Gastric\|Tumor, Colorectal | Biological: Tumor Specific Antigen-loaded Dendritic Cells\|Drug: Cyclophosphamide | Not Applicable |
| 10 | NCT03425773 | Safety and Tolerability Evaluation Study of BVAC-B in Patients With HER2/Neu(Human Epithelial Growth Factor Receptor 2) Positive Gastric Cancer After Failure to Standard Care | Completed | Stomach Neoplasms | Biological: BVAC-B | Phase 1 |
| 11 | NCT03393416 | A Phase I Clinical Study for Evaluating the Safety of MASCT-I in Advanced Soild Tumor | Unknown status | Advanced Gastric Cancer | Biological: MASCT-I\|Drug: PD1 antibody | Phase 1 |
| 12 | NCT00004604 | Biological Therapy in Treating Patients With Metastatic Cancer | Completed | Breast Cancer\|Colorectal Cancer\|Extrahepatic Bile Duct Cancer\|Gallbladder Cancer\|Gastric Cancer\|Head and Neck Cancer\|Liver Cancer\|Lung Cancer\|Metastatic Cancer\|Ovarian Cancer\|Pancreatic Cancer\|Testicular Germ Cell Tumor | Biological: CEA RNA-pulsed DC cancer vaccine | Phase 1 |
| 13 | NCT04567069 | Safety and Efficacy Study for MG-7-DC Vaccine in Gastric Cancer Treatment | Unknown status | Gastric Cancer | Biological: DC vaccine\|Biological: CTL\|Drug: Sintilimab Injection | Phase 1\|Phase 2 |
| 14 | NCT00027534 | Vaccine Therapy in Treating Patients With Advanced or Metastatic Cancer | Completed | Breast Cancer\|Colorectal Cancer\|Gallbladder Cancer\|Gastric Cancer\|Head and Neck Cancer\|Liver Cancer\|Ovarian Cancer\|Pancreatic Cancer\|Testicular Germ Cell Tumor | Biological: TRICOM-CEA(6D) | Phase 1 |
| 15 | NCT04147078 | Personalized DC Vaccine for Postoperative Cancer | Recruiting | Gastric Cancer\|Hepatocellular Carcinoma\|Non-Small-Cell Lung Cancer\|Colon Rectal Cancer | Biological: DC vaccine subcutaneous administration | Phase 1 |
| 16 | NCT01522820 | Vaccine Therapy With or Without Sirolimus in Treating Patients With NY-ESO-1 Expressing Solid Tumors | Completed | Anaplastic Astrocytoma\|Anaplastic Oligoastrocytoma\|Anaplastic Oligodendroglioma\|Estrogen Receptor Negative\|Estrogen Receptor Positive\|Glioblastoma\|Hormone-Resistant Prostate Cancer\|Metastatic Prostate Carcinoma\|Metastatic Renal Cell Cancer\|Recurrent Adult Brain Neoplasm\|Recurrent Bladder Carcinoma\|Recurrent Breast Carcinoma\|Recurrent Colorectal Carcinoma\|Recurrent Esophageal Carcinoma\|Recurrent Gastric Carcinoma\|Recurrent Hepatocellular Carcinoma\|Recurrent Lung Carcinoma\|Recurrent Melanoma\|Recurrent Ovarian Carcinoma\|Recurrent Prostate Carcinoma\|Recurrent Renal Cell Carcinoma\|Recurrent Uterine Corpus Carcinoma\|Resectable Hepatocellular Carcinoma\|Sarcoma\|Stage IA Breast Cancer\|Stage IA Ovarian Cancer\|Stage IA Uterine Corpus Cancer\|Stage IB Breast Cancer\|Stage IB Ovarian Cancer\|Stage IB Uterine Corpus Cancer\|Stage IC Ovarian Cancer\|Stage II Uterine Corpus Cancer\|Stage IIA Breast Cancer\|Stage IIA Lung Carcinoma\|Stage IIA Ovarian Cancer\|Stage IIB Breast Cancer\|Stage IIB Esophageal Cancer\|Stage IIB Lung Carcinoma\|Stage IIB Ovarian Cancer\|Stage IIB Skin Melanoma\|Stage IIC Ovarian Cancer\|Stage IIC Skin Melanoma\|Stage IIIA Breast Cancer\|Stage IIIA Esophageal Cancer\|Stage IIIA Lung Carcinoma\|Stage IIIA Ovarian Cancer\|Stage IIIA Skin Melanoma\|Stage IIIA Uterine Corpus Cancer\|Stage IIIB Breast Cancer\|Stage IIIB Esophageal Cancer\|Stage IIIB Ovarian Cancer\|Stage IIIB Skin Melanoma\|Stage IIIB Uterine Corpus Cancer\|Stage IIIC Breast Cancer\|Stage IIIC Esophageal Cancer\|Stage IIIC Ovarian Cancer\|Stage IIIC Skin Melanoma\|Stage IIIC Uterine Corpus Cancer\|Stage IV Bladder Urothelial Carcinoma\|Stage IV Esophageal Cancer\|Stage IV Ovarian Cancer\|Stage IV Prostate Cancer\|Stage IV Skin Melanoma\|Stage IVA Uterine Corpus Cancer\|Stage IVB Uterine Corpus Cancer | Biological: DEC-205/NY-ESO-1 Fusion Protein CDX-1401\|Other: Laboratory Biomarker Analysis\|Other: Pharmacological Study\|Drug: Sirolimus | Phase 1 |
| 17 | NCT02215837 | Study of Autologous Tumor Lysate-pulsed D-CIK Combined With Chemotherapy for Gastric Cancer | Unknown status | Gastric Cancer\|Neoplasms\|Gastrointestinal Neoplasms\|Digestive System Neoplasms\|Gastrointestinal Diseases | Drug: Chemotherapy\|Biological: Ag-D-CIK | Phase 2 |
| 18 | NCT03329950 | A Study of CDX-1140 (CD40) as Monotherapy or in Combination in Patients With Advanced Malignancies | Active, not recruiting | Melanoma\|Non-small Cell Lung Cancer\|Breast Cancer\|Gastric Cancer\|Renal Cell Carcinoma\|Ovarian Cancer\|Cholangiocarcinoma\|Bladder Urothelial Carcinoma\|Pancreatic Adenocarcinoma\|Colorectal Cancer\|Esophageal Cancer\|Hepatic Cancer\|Head and Neck Cancer\|Primary Peritoneal Cancer\|Fallopian Tube Cancer\|Other Solid Tumors\|Diffuse Large B-cell Lymphoma (DLBCL)\|Mantle Cell Lymphoma\|Indolent B-cell Lymphomas\|Non-Hodgkin Lymphoma\|Follicular Lymphoma\|Lymphoplasmacytic Lymphoma\|Waldenstrom's Disease\|Marginal Zone Lymphoma\|Mucosa Associated Lymphoid Tissue\|Small Lymphocytic Leukemia | Drug: CDX-1140\|Drug: CDX-301\|Drug: Pembrolizumab\|Drug: Chemotherapy | Phase 1 |
| 19 | NCT00185874 | Phase I Intratumoral Dendritic Cell Immunotherapy in Thermally Ablated Liver Metastases | Terminated | Liver Cancer | Biological: Intratumoral Dendritic Cell Immunotherapy\|Biological: autologous dendritic cells | Phase 1 |
| 20 | NCT03942328 | Modified Immune Cells (Autologous Dendritic Cells) and a Vaccine (Prevnar) After High-Dose External Beam Radiation Therapy in Treating Patients With Unresectable Liver Cancer | Recruiting | Stage III Hepatocellular Carcinoma AJCC v8\|Stage III Intrahepatic Cholangiocarcinoma AJCC v8\|Stage IIIA Hepatocellular Carcinoma AJCC v8\|Stage IIIA Intrahepatic Cholangiocarcinoma AJCC v8\|Stage IIIB Hepatocellular Carcinoma AJCC v8\|Stage IIIB Intrahepatic Cholangiocarcinoma AJCC v8\|Stage IV Hepatocellular Carcinoma AJCC v8\|Stage IV Intrahepatic Cholangiocarcinoma AJCC v8\|Stage IVA Hepatocellular Carcinoma AJCC v8\|Stage IVB Hepatocellular Carcinoma AJCC v8\|Unresectable Hepatocellular Carcinoma\|Unresectable Intrahepatic Cholangiocarcinoma | Radiation: External Beam Radiation Therapy\|Biological: Pneumococcal 13-valent Conjugate Vaccine\|Biological: Therapeutic Autologous Dendritic Cells | Early Phase 1 |
| 21 | NCT00022334 | Vaccine Therapy in Treating Patients With Liver Cancer | Completed | Liver Cancer | Biological: AFP | Phase 1\|Phase 2 |
| 22 | NCT02632188 | Radical Surgery Followed by Immunotherapy Using Precision T Cells Specific to Multiple Common Tumor-Associated Antigen for the Treatment of Hepatocellular Carcinoma | Unknown status | Liver Cancer | Procedure: Postoperative routine treatment\|Biological: DC-PMAT treatment | Phase 1\|Phase 2 |
| 23 | NCT04912765 | Neoantigen Dendritic Cell Vaccine and Nivolumab in HCC and Liver Metastases From CRC | Recruiting | Hepatocellular Carcinoma\|Hepatocellular Cancer\|Colorectal Cancer\|Colorectal Carcinoma\|Liver Metastases | Biological: Neoantigen Dendritic Cell Vaccine\|Drug: Nivolumab | Phase 2 |
| 24 | NCT02416635 | The Detection of Circulating Tumor Cells (CTCs) in Patients With Liver Cancer Undergoing Cryosurgery Combined With DC-CIK Treatment | Completed | Neoplastic Cells, Circulating | Other: Flow cytometry (FCM)\|Other: RT-PCR | |
| 25 | NCT01348256 | Study With Dendritic Cell Immunotherapy in Resected Hepatic Metastasis of Colorectal Carcinoma | Unknown status | Colorectal Carcinoma\|Hepatic Metastasis | Drug: Dendritic cells vaccine | Phase 2 |
| 26 | NCT01882946 | Safety and Efficacy Study of DCVax-Direct in Solid Tumors | Unknown status | Locally Advanced Tumor\|Metastatic Solid Tissue Tumors\|Liver Cancer\|Colorectal Cancer\|Pancreatic Cancer\|Melanoma | Biological: DCVax-Direct | Phase 1\|Phase 2 |
| 27 | NCT03889093 | Radioembolization of Primary and Secondary Liver Malignancies and The Effect On The Immune System | Recruiting | Hepatocellular Carcinoma\|Secondary Malignant Neoplasm of Liver | Other: Yttrium-90 | Not Applicable |
| 28 | NCT04476641 | A Study of DC-CIK Immunotherapy in the Treatment of Solid Tumors | Recruiting | Liver Cancer\|Kidney Cancer\|Nasopharyngeal Cancer\|Lung Cancer\|Colorectal Cancer\|Breast Cancer | Other: CELL | Phase 2 |
| 29 | NCT00610389 | Phase II Study With Immunotherapy With Dendritic Cells and Tumor Infiltrating Lymphocytes in Solid Tumors | Unknown status | Renal Cell Carcinoma\|Melanoma\|Carcinoma, Hepatocellular | Biological: immunotherapy with dendritic cells | Phase 2 |
| 30 | NCT03149523 | Exhaustive Genetic and Immunological Characterization of Colon, Kidney and Liver Tumors | Unknown status | Colorectal Adenocarcinoma\|Hepatic Carcinoma\|Kidney Adenocarcinoma | |  |
| 31 | NCT04317248 | "Cocktail" Therapy for Hepatitis B Related Hepatocellular Carcinoma | Recruiting | Hepatocellular Carcinoma | Drug: Cyclophosphamide\|Biological: Multiple Signals loaded Dendritic Cells Vaccine | Phase 2 |
| 32 | NCT02026362 | Multiple Antigen Specific Cell Therapy (MASCT) for Hepatocellular Carcinoma(HCC) Patients After Radical Resection or Radio Frequency Ablation(RFA). | Suspended | Hepatocellular Carcinoma | Biological: MASCT:Multiple Antigens Specific Cellular Therapy\|Other: The foundation treatment including against hepatitis b virus treatment using nucleoside analogue drug and protect liver treatment | Phase 1\|Phase 2 |
| 33 | NCT01128803 | Immunotherapy of Hepatocellular Carcinoma by Induction of Anti-alpha Fetoprotein Response | Terminated | Hepatocellular Carcinoma | Procedure: injection of the cell therapy product | Phase 1\|Phase 2 |
| 34 | NCT00327496 | Cytotoxicity Induced by Tumor Lysate Pulsed Dendritic Cells Against Autologous Hepatocellular Carcinoma Cells | Unknown status | Carcinoma, Hepatocellular | Biological: DC vaccine | Not Applicable |
| 35 | NCT02638857 | Immunotherapy Using Precision T Cells Specific to Multiple Common Tumor-Associated Antigen Combined With Transcatheter Arterial Chemoembolization for the Treatment of Advanced Hepatocellular Carcinoma | Unknown status | Recurrence Hepatocellular Carcinoma\|Advanced Hepatocellular Carcinoma | Procedure: TACE\|Biological: Dendritic Cell\|Drug: lipiodol\|Drug: Mitomycin (MMC)\|Drug: Epirubicin(EADM)\|Biological: Precision Multiple Antigen T Cell | Phase 1\|Phase 2 |
| 36 | NCT01974661 | Phase I Safety Study of Dendritic Cell Vaccine to Treat Patients With Hepatocellular Carcinoma | Completed | Hepatocellular Carcinoma | Biological: COMBIG-DC (ilixadencel) | Phase 1 |
| 37 | NCT00228189 | Carcinoembryonic Antigen-loaded Dendritic Cells in Advanced Colorectal Cancer Patients | Completed | Colorectal Cancer\|Liver Metastases | Biological: CEA-loaded dendritic cell vaccine | Phase 1\|Phase 2 |
| 38 | NCT03222076 | Nivolumab With or Without Ipilimumab in Treating Patients With Resectable Liver Cancer | Active, not recruiting | Hepatocellular Carcinoma\|Resectable Hepatocellular Carcinoma | Biological: Ipilimumab\|Biological: Nivolumab | Phase 2 |
| 39 | NCT02632006 | Immunotherapy Using Pluripotent Killer-Programmed Cell Death 1 (PIK-PD-1) Cells for the Treatment of Advanced Hepatocellular Carcinoma | Unknown status | Advanced Hepatocellular Carcinoma | Biological: PIK-PD-1 cells\|Biological: DC-PMAT | Phase 1\|Phase 2 |
| 40 | NCT00103142 | Vaccine Therapy in Treating Patients With Liver or Lung Metastases From Colorectal Cancer | Completed | Colorectal Cancer\|Metastatic Cancer | Biological: falimarev\|Biological: inalimarev\|Biological: sargramostim\|Biological: therapeutic autologous dendritic cells | Phase 2 |
| 41 | NCT00003433 | Immunotherapy in Treating Patients With Resected Liver Metastases From Colon Cancer | Completed | Colorectal Cancer\|Metastatic Cancer | Biological: carcinoembryonic antigen RNA-pulsed DC cancer vaccine | Phase 1\|Phase 2 |
| 42 | NCT02281266 | Thymalfasin Adjuvant Therapy in Hepatitis B Virus (HBV)-Related Hepatocellular Carcinoma (HCC) After Curative Resection | Unknown status | Curable Hepatitis B Virus-Related Hepatocellular Carcinoma | Procedure: curative resection\|Drug: thymalfasin\|Drug: nucleoside analog (suggest to use entecavir) | Phase 4 |
| 43 | NCT04380545 | Nivolumab, Fluorouracil, and Interferon Alpha 2B for the Treatment of Unresectable Fibrolamellar Cancer | Recruiting | Stage III Hepatocellular Carcinoma AJCC v8\|Stage IIIA Hepatocellular Carcinoma AJCC v8\|Stage IIIB Hepatocellular Carcinoma AJCC v8\|Stage IV Hepatocellular Carcinoma AJCC v8\|Stage IVA Hepatocellular Carcinoma AJCC v8\|Stage IVB Hepatocellular Carcinoma AJCC v8\|Unresectable Fibrolamellar Carcinoma | Drug: Fluorouracil\|Biological: Nivolumab\|Biological: Recombinant Interferon Alpha 2b-like Protein | Phase 1\|Phase 2 |
| 44 | NCT01821482 | A Study of DC-CIK to Treat Hepatocellular Carcinoma | Unknown status | Hepatocellular Carcinoma (HCC) | Biological: Dendritic and Cytokine-induced Killer Cells | Phase 2 |
| 45 | NCT02632019 | Immunotherapy Using Precision T Cells Specific to Personalized Neo-antigen for the Treatment of Advanced Malignant Tumor of Biliary Tract | Unknown status | Advanced Biliary Tract Malignant Tumor | Drug: Gemcitabine\|Biological: Dendritic cell-precision T cell for neo-antigen combined with gemcitabine treatment | Phase 1\|Phase 2 |
| 46 | NCT03358849 | Phase 1 Clinical Trial to Evaluate the Safety of Allogeneic NK Cell ("SMT-NK") Cell Therapy in Advanced Biliary Tract Cancer | Completed | Advanced Biliary Tract Cancer | Biological: Natural killer cell | Phase 1 |
| 47 | NCT04004234 | A Phase I/II Study of the Pan-immunotherapy in Patients With Local Advanced/Metastatic BTC | Unknown status | Biliary Tract Cancer (BTC) | Drug: Manganese Chloride\|Drug: nab-paclitaxel\|Drug: Gemcitabine\|Drug: anti-PD-1 antibody | Phase 1\|Phase 2 |
| 48 | NCT04969887 | Combination Immunotherapy in Rare Cancers Under InvesTigation | Recruiting | Advanced Biliary Tract Cancer\|Neuroendocrine Tumors\|Female Reproductive System Neoplasm\|MSI-H Solid Malignant Tumor | Drug: Ipilimumab\|Drug: Nivolumab | Phase 2 |
| 49 | NCT03214939 | Autologous Antigen-activated Dendritic Cells in the Treatment of Patients With Colorectal Cancer | Unknown status | Colorectal Cancer | Biological: Immunotherapy based on dendritic cells | Early Phase 1 |
| 50 | NCT00154713 | Immunotherapy for Colorectal Cancers Using CEA-Pulsed Dendritic Cells and Subsequent IL-2 Treatment | Unknown status | Colorectal Cancer | Biological: CEA pulsed dendritic cells | Phase 1\|Phase 2 |
| 51 | NCT02503150 | Trial of Antigen Pulsed Dendritic Cells (APDC) in Metastatic Colorectal Cancer | Unknown status | Metastatic Colorectal Cancer | Biological: APDC + Chemotherapy\|Drug: Chemotherapy | Phase 3 |
| 52 | NCT02919644 | Vaccination With Autologous Dendritic Cells Loaded With Autologous Tumour Homogenate After Curative Resection for Stage IV Colorectal Cancer. | Recruiting | Stage IV Colorectal Cancer\|Curative Resection | Biological: autologous dendritic cells loaded with autologous tumour homogenate\|Drug: IL2 | Phase 2 |
| 53 | NCT03152565 | Avelumab Plus Autologous Dendritic Cell Vaccine in Pre-treated Metastatic Colorectal Cancer Patients | Completed | Colorectal Carcinoma | Drug: Avelumab | Phase 1\|Phase 2 |
| 54 | NCT02615574 | A Study of Type-1 Polarized Dendritic Cell (伪DC1) Vaccine in Combination With Tumor-Selective Chemokine Modulation (Interferon-伪2b, Rintatolimod, and Celecoxib) in Subjects With Chemo-Refractory Metastatic Colorectal Cancer | Withdrawn | Metastatic Colorectal Cancer | Biological: 伪DC1 vaccine\|Drug: CKM | Phase 2 |
| 55 | NCT00558051 | Alpha-type-1 Dendritic Cell-based Vaccines in Patients With Metastatic Colorectal Cancer | Completed | Metastatic Colorectal Cancer | Biological: DC-based vaccine | Phase 1 |
| 56 | NCT01885702 | Dendritic Cell Vaccination in Patients With Lynch Syndrome or Colorectal Cancer With MSI | Active, not recruiting | Colorectal Cancer | Biological: DC vaccination | Phase 1\|Phase 2 |
| 57 | NCT05518032 | Pembrolizumab and Autologous Dendritic Cells for the Treatment of Refractory Colorectal Cancer (CRC) | Not yet recruiting | Metastatic Microsatellite Stable Colorectal Carcinoma\|Recurrent Colorectal Carcinoma\|Stage III Colorectal Cancer AJCC v8\|Stage IV Colorectal Cancer AJCC v8\|Unresectable Colorectal Carcinoma | Procedure: Biopsy\|Biological: Pembrolizumab\|Biological: Therapeutic Autologous Dendritic Cells | Phase 2 |
| 58 | NCT00176761 | Tumor-Pulsed Dendritic Cells Used as a Tumor Vaccine | Terminated | Metastatic Colorectal Cancer | Drug: Interleukin-2 (IL-2) | Phase 2 |
| 59 | NCT03730948 | DC Vaccine in Colorectal Cancer | Recruiting | Colorectal Cancer | Biological: DC vaccine | Phase 1 |
| 60 | NCT01413295 | Randomized Trial With Dendritic Cells in Patients With Metastatic Colorectal Cancer | Completed | Colorectal Neoplasms | Drug: Dendritic Cell Vaccine\|Other: Supportive treatment | Phase 2 |
| 61 | NCT00311272 | Dendritic Cell Vaccination in Patients With Advanced Colorectal Cancer | Completed | Colorectal Neoplasms | Biological: MelCancerVac | Phase 2 |
| 62 | NCT01671592 | Safety of Labeled Dendritic Cell (DC) Vaccines and Feasibility of Tracking by Magnetic Resonance Imaging (MRI) | Completed | Colorectal Neoplasms\|Colorectal Cancer\|Colorectal Carcinoma\|Colorectal Tumors\|Neoplasms, Colorectal | Biological: DC Vaccine | Phase 1 |
| 63 | NCT00019591 | Vaccine Therapy With or Without Interleukin-2 in Treating Patients With Locally Advanced or Metastatic Colorectal Cancer | Completed | Colorectal Cancer | Biological: aldesleukin\|Biological: ras peptide cancer vaccine\|Procedure: adjuvant therapy | Phase 1\|Phase 2 |
| 64 | NCT02450422 | The Detection of Circulating Tumor Cells (CTCs) in Patients With Colorectal Cancer Undergoing Cryosurgery Combined With DC-CIK Treatment | Completed | Neoplastic Cells, Circulating | Other: Flow cytometry (FCM)\|Other: RT-PCR | |
| 65 | NCT03202758 | Evaluation of the Safety and the Tolerability of Durvalumab Plus Tremelimumab Combined With FOLFOX in mCRC | Unknown status | Colorectal Cancer Metastatic | Drug: Durvalumab, Tremelimumab and ,FOLFOX | Phase 1\|Phase 2 |
| 66 | NCT03193710 | The Effects of General Anesthetics on Lymphocytes in Patients Undergoing Colorectal Cancer Resection and Mechanism Involved | Recruiting | Colorectal Cancer Metastatic\|Survival Rate\|General Anesthetics Toxicity\|Lymphocyte Destruction\|Molecular Mechanism of Pharmacological Action | Drug: Propofol\|Drug: Sevoflurane\|Drug: Remifentanil | |
| 67 | NCT00128622 | Denileukin Diftitox Followed by Vaccine Therapy in Treating Patients With Metastatic Cancer | Completed | Breast Cancer\|Colorectal Cancer\|Lung Cancer\|Pancreatic Cancer\|Unspecified Adult Solid Tumor, Protocol Specific | Biological: denileukin diftitox\|Biological: recombinant fowlpox-CEA(6D)/TRICOM vaccine\|Biological: therapeutic autologous dendritic cells | Phase 1 |
| 68 | NCT03854799 | Immunotherapy In Locally Advanced Rectal Cancer | Active, not recruiting | Colon Rectal Cancer | Drug: Avelumab\|Drug: Capecitabine\|Radiation: EXTERNAL---BEAM IRRADIATION 50.4 GY | Phase 2 |
| 69 | NCT00780988 | Feasibility of Autologous Tumor Cell-TLR9 Agonist Vaccination for Metastatic Colorectal Cancer | Withdrawn | Colorectal Neoplasms\|Anal, Colon, and Rectal Cancers | Biological: Autologous tumor cell + CpG vaccine\|Procedure: Autologous hematopoietic and immune cell rescue (transplantation) | Phase 1 |
| 70 | NCT04438564 | Immunoassay and Regulation of Traditional Chinese Medicine on Cancer Patients | Recruiting | Breast Cancer\|Colorectal Cancer\|Cancer of Ovary\|Cancer of Endometrium | Other: Cases for patients diagnosed with breast cancer, colonrectal cancer, ovarian cancer and endometrial cancer | Not Applicable |
| 71 | NCT00936572 | Probiotics In Colorectal Cancer Patients | Completed | Colorectal Cancer | Procedure: Probiotics (La1, BB536)\|Biological: probiotics (La1, BB536)\|Biological: placebo | Phase 2 |
| 72 | NCT02886897 | A Study of Combinations of D-CIK Immunotherapy And Anti-PD-1 In Refractory Solid Tumors | Unknown status | Hepatocellular Carcinoma\|Renal Cell Carcinoma\|Bladder Cancer\|Colorectal Cancer\|Non-small-cell Lung Cancer\|Breast Cancer | Biological: D-CIK and anti-PD-1 antibody | Phase 1\|Phase 2 |
| 73 | NCT00648102 | Phase I Study of CDX-1307, hCG-B Vaccine, for Patients With Incurable, Locally Advanced or Metastatic Breast, Colorectal, Pancreatic, Bladder or Ovarian Cancer | Completed | Breast Cancer\|Colorectal Cancer\|Pancreatic Cancer\|Bladder Cancer\|Ovarian Cancer | Biological: CDX-1307 | Phase 1 |
| 74 | NCT01839539 | Study of DC-CIK to Treat Colorectal Cancer | Unknown status | Colorectal Cancer | Biological: dendritic and cytokine-induced killer cells | Phase 2 |
| 75 | NCT04001101 | Anti-PD-1 +/- RT for MSI-H Solid Tumors | Withdrawn | Microsatellite Instability High\|Mismatch Repair Deficiency\|Colorectal Cancer | Combination Product: RT and Anti-PD-1\|Drug: Anti-PD-1 | Phase 2 |
| 76 | NCT00019084 | Vaccine Therapy and Biological Therapy in Treating Patients With Advanced Cancer | Completed | Breast Cancer\|Cervical Cancer\|Colorectal Cancer\|Lung Cancer\|Ovarian Cancer\|Pancreatic Cancer | Biological: aldesleukin\|Biological: mutant p53 peptide pulsed dendritic cell vaccine\|Biological: ras peptide cancer vaccine\|Biological: sargramostim\|Biological: therapeutic autologous lymphocytes\|Biological: therapeutic tumor infiltrating lymphocytes | Phase 2 |
| 77 | NCT02202928 | Adoptive Cell Therapy Plus Chemotherapy and Radiation After Surgery in Treating Patients With Colorectal Cancer | Unknown status | Colorectal Cancer\|Neoplasms\|Intestinal Neoplasms\|Digestive System Neoplasms\|Gastrointestinal Diseases | Biological: DC-CIK\|Radiation: Radiotherapy\|Drug: Chemotherapy | Phase 2 |
| 78 | NCT03185429 | TSA-DC Vaccine in Treating Patients With Gastrointestinal Solid Tumor | Unknown status | Tumor Gastric\|Tumor, Colorectal | Biological: Tumor Specific Antigen-loaded Dendritic Cells\|Drug: Cyclophosphamide | Not Applicable |
| 79 | NCT03047525 | Study of DC-CTL Combined With CIK for Advanced Solid Tumor | Unknown status | Colorectal Cancer\|Renal Cell Carcinoma\|Nasopharyngeal Carcinoma\|Lung Cancer | Biological: Cytokine-induced Killer Cells | Phase 1\|Phase 2 |
| 80 | NCT02298959 | Testing the PD-1 Antibody, MK3475, Given With Ziv-aflibercept in Patients With Advanced Cancer | Active, not recruiting | Clinical Stage IV Cutaneous Melanoma AJCC v8\|Metastatic Colorectal Carcinoma\|Metastatic Malignant Solid Neoplasm\|Metastatic Melanoma\|Metastatic Ovarian Carcinoma\|Metastatic Renal Cell Carcinoma\|Pathologic Stage IV Cutaneous Melanoma AJCC v8\|Platinum-Resistant Ovarian Carcinoma\|Recurrent Melanoma\|Recurrent Renal Cell Carcinoma\|Refractory Melanoma\|Refractory Renal Cell Carcinoma\|Sarcoma\|Stage IV Colorectal Cancer AJCC v8\|Stage IV Ovarian Cancer AJCC v8\|Stage IV Renal Cell Cancer AJCC v8\|Stage IVA Colorectal Cancer AJCC v8\|Stage IVA Ovarian Cancer AJCC v8\|Stage IVB Colorectal Cancer AJCC v8\|Stage IVB Ovarian Cancer AJCC v8\|Stage IVC Colorectal Cancer AJCC v8 | Biological: Pembrolizumab\|Biological: Ziv-Aflibercept | Phase 1 |
| 81 | NCT02882659 | Dendritic Killer Cell-based Immunotherapy for Solid Tumors | Unknown status | Colorectal Neoplasms\|Hepatocellular Carcinoma\|Neoplasm Metastasis | Biological: Dendritic Killer Cell (DKC) | Phase 1 |
